# Chloroquine reduces hypercoagulability in pancreatic cancer through inhibition of neutrophil extracellular traps

**DOI:** 10.1186/s12885-018-4584-2

**Published:** 2018-06-22

**Authors:** Brian A. Boone, Pranav Murthy, Jennifer Miller-Ocuin, W. Reed Doerfler, Jarrod T. Ellis, Xiaoyan Liang, Mark A. Ross, Callen T. Wallace, Jason L. Sperry, Michael T. Lotze, Matthew D. Neal, Herbert J. Zeh

**Affiliations:** 1 Department of Surgery, University of Pittsburgh, Pittsburgh, PA USA; 2 Center for Biologic Imaging, University of Pittsburgh, Pittsburgh, PA USA; 3 Departments of Thoracic Surgery, University of Pittsburgh, Pittsburgh, PA USA; 4 Immunology, University of Pittsburgh, Pittsburgh, PA USA; 5 Bioengineering, University of Pittsburgh, Pittsburgh, PA USA; 6 UPMC Cancer Pavilion, University of Pittsburgh, Suite 417, 5150 Centre Ave, Pittsburgh, PA 15232 USA

**Keywords:** Chloroquine, Autophagy, Neutrophil extracellular traps (NETs), Hypercoagulability, Venous thromboembolism

## Abstract

**Background:**

The hypercoagulable state associated with pancreatic adenocarcinoma (PDA) results in increased risk of venous thromboembolism, leading to substantial morbidity and mortality. Recently, neutrophil extracellular traps (NETs), whereby activated neutrophils release their intracellular contents containing DNA, histones, tissue factor, high mobility group box 1 (HMGB1) and other components have been implicated in PDA and in cancer-associated thrombosis.

**Methods:**

Utilizing an orthotopic murine PDA model in C57/Bl6 mice and patient correlative samples, we studied the role of NETs in PDA hypercoagulability and targeted this pathway through treatment with the NET inhibitor chloroquine. PAD4 and RAGE knockout mice, deficient in NET formation, were used to study the role of NETs in platelet aggregation, release of tissue factor and hypercoagulability. Platelet aggregation was assessed using collagen-activated impedance aggregometry. Levels of circulating tissue factor, the initiator of extrinsic coagulation, were measured using ELISA. Thromboelastograms (TEGs) were performed to assess hypercoagulability and changes associated with treatment. Correlative data and samples from a randomized clinical trial of preoperative gemcitabine/nab-paclitaxel with and without hydroxychloroquine were studied and the impact of treatment on venous thromboembolism (VTE) rate was evaluated.

**Results:**

The addition of NETs to whole blood stimulated platelet activation and aggregation. DNA and the receptor for advanced glycation end products (RAGE) were necessary for induction of NET associated platelet aggregation. PAD4 knockout tumor-burdened mice, unable to form NETs, had decreased aggregation and decreased circulating tissue factor. The NET inhibitor chloroquine reduces platelet aggregation, reduces circulating tissue factor and decreases hypercoagulability on TEG. Review of correlative data from patients treated on a randomized protocol of preoperative chemotherapy with and without hydroxychloroquine demonstrated a reduction in peri-operative VTE rate from 30 to 9.1% with hydroxychloroquine that neared statistical significance (*p* = 0.053) despite the trial not being designed to study VTE.

**Conclusion:**

NETs promote hypercoagulability in murine PDA through stimulation of platelets and release of tissue factor. Chloroquine inhibits NETs and diminishes hypercoagulability. These findings support clinical study of chloroquine to lower rates of venous thromboembolism in patients with cancer.

**Trial registration:**

This study reports correlative data from two clinical trials that registered with clinicaltrials.gov, NCT01128296 (May 21, 2010) and NCT01978184 (November 7, 2013).

**Electronic supplementary material:**

The online version of this article (10.1186/s12885-018-4584-2) contains supplementary material, which is available to authorized users.

## Background

Pancreatic cancer is associated with a hypercoagulable state resulting in a high risk of venous thromboembolism (VTE), which affects up to 40% of patients during their course of disease [1–3]. Development of VTE in patients with pancreatic cancer is associated with a poor prognosis [4, 5]. Despite various approaches for thromboprophylaxis, both VTE and subsequent treatments for it are significant sources of morbidity and mortality. Novel pathways and therapeutic approaches to prevent VTE events are needed [6].

A recently described phenomenon that occurs in activated neutrophils, neutrophil extracellular trap formation or NETs, has been described as a potential contributor to hypercoagulability. NETs have been linked to thrombosis in autoimmune conditions and sterile inflammation [7, 8] and more recently implicated in cancer associated thrombosis [9–11]. Neutrophil extracellular traps (NETs) occur when activated neutrophils release their intracellular contents, including DNA, histones, granules and proteins, into the surrounding tissue or circulation [12]. We have previously demonstrated that pancreatic cancer primes neutrophils to become more prone to NET formation and identified NETs within pancreatic tumors [13].

Autophagy, a cancer cell survival mechanism whereby damaged organelles, proteins and other intracellular components are recycled, appears to be critical for NET formation in pancreatic cancer [13]. Furthermore, the autophagy inhibitor chloroquine inhibits NET formation [13, 14]. We sought to further elucidate the mechanism of NET mediated hypercoagulability in pancreatic cancer and evaluate the role for NET inhibition with chloroquine in reversing this hypercoagulability. NETs and down-stream signaling pathways represent a novel target for further research on cancer associated thrombosis [15].

## Methods

### Murine studies and treatments

All experimental procedures were reviewed and approved by the Institutional Animal Care and Use Committee of the University of Pittsburgh (Protocol # 14084123) and performed in accordance with the guidelines established by the University of Pittsburgh Division of Laboratory Animal Services and the American Veterinary Medical Association and in accordance with the Guide for the Care and Use of Laboratory Animals. Euthanasia was performed using CO_2_ inhalation or under the surgical plane of anesthesia via cardiac puncture resulting in exsanguination followed by cervical dislocation. Mice were housed in ventilated caging units in the Hillman Cancer Center Specific Pathogen Free (SPF) facility with standard housing and husbandry and free access to food and water.

C57/Bl6 wild-type mice (10–12-week female weighing 20–30 g) were purchased from Taconic (Hudson, NY, USA). Mice genetically deficient in protein arginine deiminase 4 (PAD4 KO), an enzyme required for NET formation were a generous gift from the late Kerri Mowen (Scripps Institute). The generation of these mice from a C57/Bl6 background has been previously described [16]. Knockout mice deficient in the receptor for advanced glycation end products (RAGE−/−, SVEV129 x C57/BL6), a critical inducer of autophagy and NET formation in pancreatic cancer, were also studied and made available by the late Angelika Bierhaus (Heidelberg). For the orthotopic pancreatic cancer model, wild type, RAGE KO and PAD4 KO mice were randomly allocated and injected with 1 × 10^6^ Panc02 cells (National Cancer Institute repository, 2008) into the tail of the pancreas through a limited laparotomy. Anesthesia was induced using isoflurane (2–5% inhalation), ketamine (90 mg/kg IP) and xylazine (10 mg/kg IP). Buprenex (0.1 mg/kg IP BID for 3 days) was administered for postoperative pain control. Animals were sacrificed 4 weeks following injection at which time they had palpable left upper quadrant abdominal tumors. Prior to injection, cells were cultured in RPMI 1640 media (Hyclone, Logan, UT, USA) with 10% fetal bovine serum, and PenStrep antibiotic (Gemini, West Sacramento, CA, USA) in a humidified incubator with 5% CO^2^. Mice were treated with oral chloroquine administered in the drinking water (0.5 mg/mL, MP Biomedicals, Solon, OH, USA). Mice were treated with DNase I (Sigma Aldrich, St Louis, MO, USA) for 5 consecutive daily intraperitoneal injections (5 mg/kg) prior to sacrifice. The n for each experiment reports the number of individual animals.

### Ex vivo neutrophil extracellular trap formation

Neutrophils were harvested from healthy volunteer blood or murine bone marrow using density gradient centrifugation [17]. Cells were initially plated in Hank’s Balanced Salt Solution (HBSS, Gibco, Grand Island, NY, USA), then to form NETs, HBSS was removed and cells were stimulated with 500 nM phorbol 12-myristate 13-acetate (PMA, Sigma, St. Louis, MO, USA) in RPMI. Supernatant was collected after 4 h and the formation of NETs was confirmed by measuring supernatant DNA using Quant-iT Picogreen (Invitrogen, Grand Island, NY, USA, MP07581) and by fluorescence microscopy to visualize NET formation using DNA staining with Hoechst (Additional file 1: Figure S1).

### Platelet activation and aggregation

Platelet activation was assessed by analyzing expression of P-selectin (CD62P) by flow cytometry using an APC-conjugated anti-CD62P monoclonal antibody (2 μg/ml, mouse IgG1κ; eBioscience, San Diego, CA) or isotype control antibody (eBioscience) in platelet rich plasma (PRP), obtained by platelet isolation centrifugation. A BD Accuri C6 Plus (BD Biosciences, San Jose, CA) flow cytometer and FlowJo software (Tree Star, Ashland, OR) were used to measure %CD62P positive platelets. Platelets were gated based on their characteristic scatter properties. Whole blood platelet aggregation was measured using impedance aggregometry (ChronoLog aggregometer, Model 700, Havertown, PA, USA). Platelets were activated with collagen (2 μg/ml; ChronoLog) and aggregation was measured for 6 min at 37 °C with a stir speed of 1200 rpm and gain of 0.01. Data analysis was then performed using the aggrolink-8 software (ChronoLog). Data is reported as the area under the curve (AUC), which incorporates both the slope and amplitude of the aggregation curve. Murine whole blood was tested after submandibular bleed or cardiac puncture into 3.4% sodium citrated with 10 units/mL heparin. Human (500 μL) and murine (300 μL) whole blood was treated with 50 to 100 μL of NET supernatant for 10 min. RPMI media with 500 nM PMA was added to whole blood for a control. 1 mg/mL treatment of DNase I (Sigma Aldrich, St. Louis, MO, USA) was added to NET supernatant for 10 min prior to treatment of whole blood. 100 μg/mL chloroquine (MP Biomedicals) was added to whole blood for 10 min prior to aggregation.

### Clinical correlative samples and trial protocols

Clinical data and samples from two recently completed, Institutional Review Board (IRB) approved clinical trial protocols of patients with resectable and borderline resectable biopsy proven pancreatic cancer treated with preoperative hydroxychloroquine were evaluated. The first trial was a dose escalation Phase I/II trial of preoperative gemcitabine with hydroxychloroquine for patients with high risk pancreatic adenocarcinoma (UPCI 09–122, IRB Protocol #10010028) [18]. A more recent trial randomized patients to two cycles of preoperative gemcitabine/nab-paclitaxel with or without 1200 mg/day oral hydroxychloroquine (UPCI 13–074, IRB Protocol #13080444). In both trials, hydroxychloroquine was initiated 48 h before the first dose of chemotherapy and continued until the day before surgery. These studies were not powered to evaluate the exploratory endpoints including in the current manuscript. Patient blood was drawn pre- and post-chemotherapy treatment. Plasma was collected from blood drawn into 3.2% sodium citrate tubes. Serum was collected after blood was allowed to clot and then spun at 1000 g for 10 min. Serum and plasma samples were stored at − 80 °C.

Resected pancreatic specimens from patients with pancreatic adenocarcinoma were stained and imaged using the following protocol. Following standard IHC deparaffinization protocol, sections were subject to antigen retrieval using 10 mM Citric acid buffer. Post antigen retrieval, sections were washed three times with phosphate buffered saline (PBS), followed by 3× washes with solution of 0.5% BSA in PBS. Sections were blocked with 5% donkey serum in BSA solution for 45 min. The slides were incubated for 1 h at room temperature (RT) with primary antibodies for rabbit anti neutrophil elastase (ab68672, Abcam) at 1:200, sheep anti fibrinogen (ab61352, Abcam) 1:1000, and mouse anti tissue factor (ab17375, Abcam) 1:200, in 0.5% BSA solution. Slides were washed three times with BSA solution and incubated for 1 h at RT with Alexa 488 donkey anti mouse secondary antibody (A21202, Invitrogen) diluted 1:500, combined with donkey anti rabbit CY3 (711–165-152, Jackson Immuno) 1:1000, and donkey anti sheep Cy5 (713–175-147, Jackson) in BSA solution. Nuclei were stained with Hoechst dye (bisbenzamide 1 mg/100 ml water) for 30 s. After three rinses with PBS, sections were cover slipped with Gelvatol mounting media. Large area scan images were captured with a Nikon A1confocal microscope (NIS Elements 4.4, Tokyo, Japan).

For clinical outcomes, venous thromboembolism was defined as any venous thrombosis including deep vein thrombosis, pulmonary embolism, mesenteric thrombosis and catheter associated thrombosis. Venous thromboembolism was reported from the initiation of treatment through the 90 day postoperative period.

### Tissue factor analysis

Serum was collected after blood was allowed to clot for 30 min and then spun at 1000 g for 10 min. A 10 fold dilution was performed and mouse tissue factor levels were measured using the F3 / CD142 / Tissue factor ELISA per the manufacturer’s instruction (LS Bio, LS-F14709, Seattle, WA, USA). The human F3/CD142/Tissue factor ELISA kit was used to measure tissue factor in patient blood samples (LS Bio, LS-F433).

### Thromboelastography (TEG)

TEG was performed on 340 μl murine whole blood drawn via submandibular bleed mixed with 1:9 dilution of 3.4% sodium citrate and 10 units/mL heparin using a Haemoscope 5000 analyzer (Haemonetics, Braintree, MA, USA) as previously described [19]. Samples were placed into TEG cups 2 IU of Heparinase I and 20 μL of 0.2 mol/l CaCl_2_ was added. Curve analysis was performed using Haemonetics TEG software (version 4.2.3) and the R, K, angle, and MA were measured. The primary outcome for hypercoagulability was the coagulation index, a value that incorporates all measurements from the TEG curve [20].

### Statistical analysis

Data are expressed as mean ± standard deviation. Results are reported from at least two independent experiments performed with at least duplicate samples. Analysis was performed by using Student’s two tailed t-test or 1-way ANOVA with Tukey’s post-hoc test using Graph Pad Prism software (GraphPad, San Diego CA, USA). Pre and post-treatment results were compared using paired t-test. *P*-values < 0.05 were considered statistically significant.

## Results

### NETs promote platelet aggregation through a DNA/RAGE dependent mechanism

**Fig. 1:**
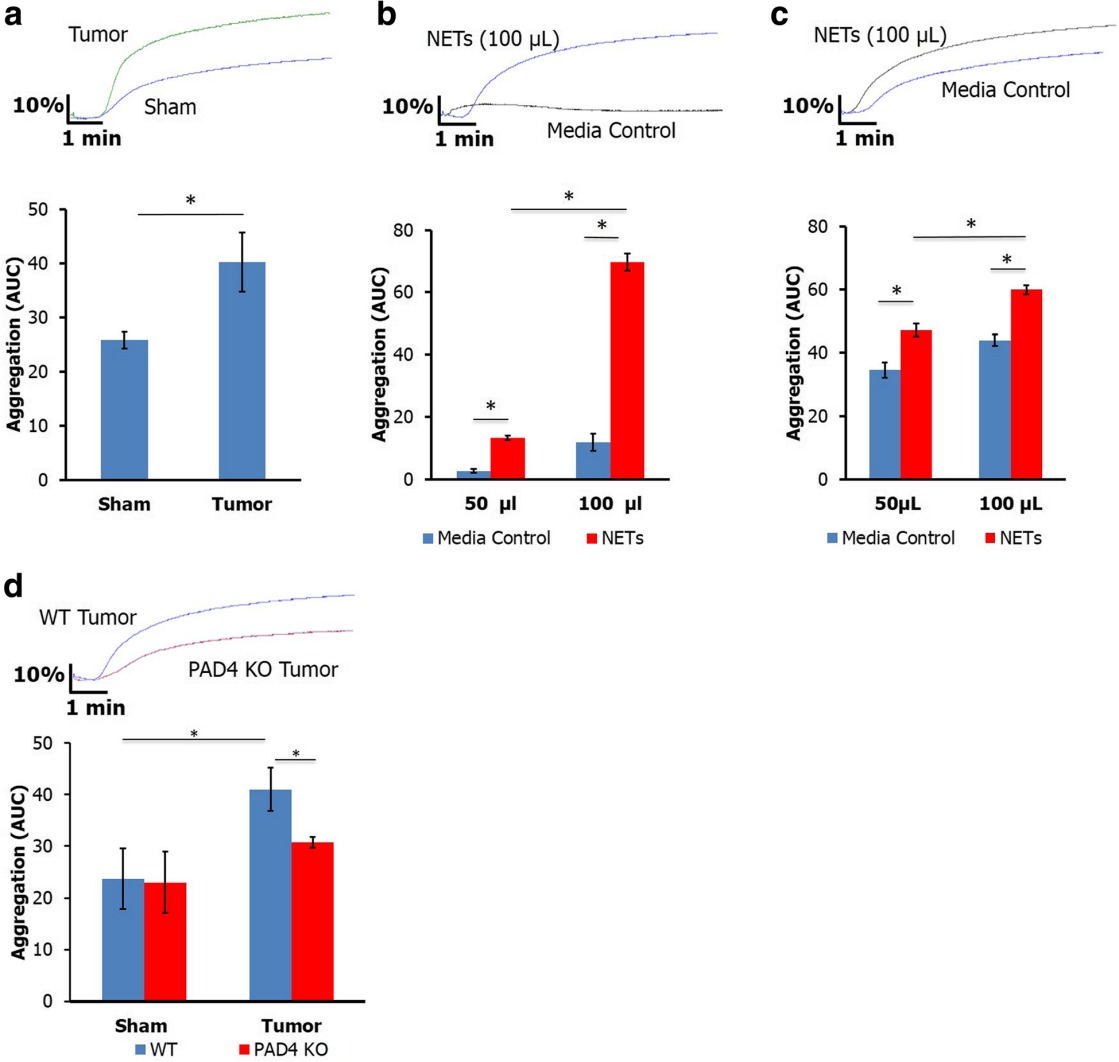
NETs promote hypercoagulability through platelet aggregation. Tumor bearing mice have elevated platelet aggregation compared with sham controls (**a**, AUC 40.2 ± 5.5 vs. 25.8 ± 1.5, *n* = 5). Treatment of human (**b**) and murine (**c**) blood with NET supernatant led to a dose dependent increase in platelet aggregation compared with treatment with media control. Tumor bearing PAD4 KO mice had decreased platelet aggregation compared to WT (AUC 8.4 ± 2.4 vs. 3.7 ± 1.7, *n* = 7) with no difference in sham controls (**d**). *p < 0.05

The interaction between NETs and platelets has been implicated in the pathogenesis of deep vein thrombosis [21]. To determine the role of NETs in platelet aggregation in our cancer model, we first examined platelet activation and aggregation in mice injected with orthotopic tumor and sham injected controls. Mice from tumor bearing animals demonstrated significantly greater platelet aggregation in response to collagen stimulation (Fig. 1a) and had heightened platelet activation as measured by %CD62P positive platelets (Additional file 1: Figure S1A). To determine if NETs played a role in this enhanced platelet function, we treated whole blood from C57/Bl6 wild type mice and healthy human volunteers with NET supernatant for 10 min and assessed platelet activation and aggregation. Treatment with NET supernatant induced platelet aggregation in both human (Fig. 1b) and murine (Fig. 1c) blood in a dose dependent fashion and increased platelet activation (Additional file 2: Figure S2B). Furthermore, staining of resected human pancreatic tumors demonstrated focal areas of neutrophil and fibrinogen conjugates (Additional file 3: Figure S3), suggesting potential interaction between neutrophils and platelets in thrombosis within the pancreatic tumor microenvironment.

To substantiate the role of NETs in upregulated platelet function, we injected orthotopic tumor into the pancreas of PAD4 KO and syngeneic wild type controls. PAD4 KO mice are unable to form NETs as a result of genetic deficiency in protein arginine deiminase 4, an enzyme critical for NET formation that citrullinates histones to allow for DNA unwinding and expulsion from the cell [22]. PAD4 KO tumor bearing mice demonstrated decreased platelet activation (Additional file 2: Figure S2A) and aggregation compared with WT tumor bearing controls (Fig. 1d). Together these findings support that enhanced platelet function in tumor bearing mice is associated with NETs.

**Fig. 2:**
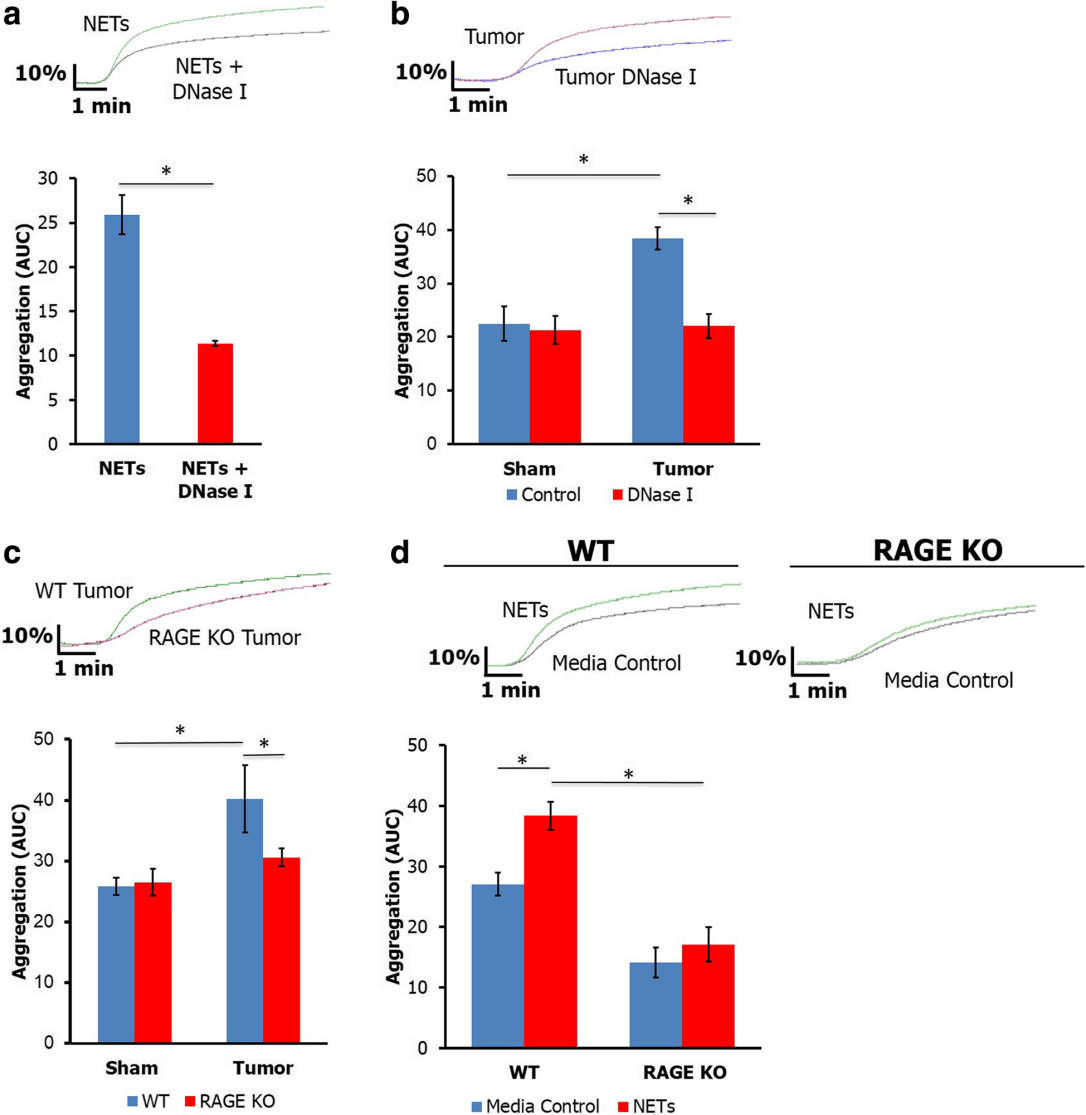
NET upregulation of platelet aggregation is mediated by neutrophil DNA and platelet RAGE. Removing DNA from NET supernatant using DNase I treatment prior to exposure to whole blood reversed the treatment effects of NET supernatant on platelet aggregation in human blood (**a**, 25.9 ± 2.2 vs. 11.35 ± 0.31, *n* = 4, *p* < 0.05). In vivo treatment with DNase I resulted in decreased aggregation in tumor bearing mice (**b**, AUC 22.1 ± 2.3 vs. 38.4 ± 2.1, *n* = 4, *p* < 0.05). Tumor bearing RAGE KO mice have decreased platelet aggregation compared to WT mice (**c**, AUC 30.6 ± 1.5 vs. 40.2 ± 5.5, *n* = 4, *p* < 0.05). Blood from RAGE knockout mice had decreased aggregation after treatment with 100 μL of NET supernatant compared with WT (**d**, AUC 25.5 ± 2.6 vs. 43.3 ± 3.9, *n* = 4, *p* < 0.05). *p < 0.05

During the formation of NETs, DNA is the principle factor released, however many other intracellular components including tissue factor, myeloperoxidase, and histones are also released. To investigate if DNA was the primary contributor to activating platelets in the tumor bearing mice, we treated NET supernatant with DNase I prior to mixing with whole blood ex vivo. Treatment of NET supernatant with DNase diminished platelet aggregation (Fig. 2a). Next, we treated tumor bearing mice with DNase I and observed a significant reduction in platelet aggregation (Fig. 2b). Because the receptor for advanced glycation end products (RAGE) is a known receptor for DNA [23] and induces autophagy and NET formation in pancreatic cancer [13], we sought to evaluate the role of RAGE in NET mediated platelet aggregation. Platelet aggregometry was performed on RAGE knockout (RAGE KO) animals, which have global genetic depletion of RAGE. RAGE KO tumor bearing mice had decreased platelet aggregation compared to WT tumor bearing mice (Fig. 2c). Furthermore, treatment of whole blood from RAGE KO mice with NET supernatant led to diminished platelet aggregation compared with WT mice (Fig. 2d). These findings implicate a role for DNA and RAGE in NET induced platelet aggregation.

### NETs increase circulating tissue factor

**Fig. 3:**
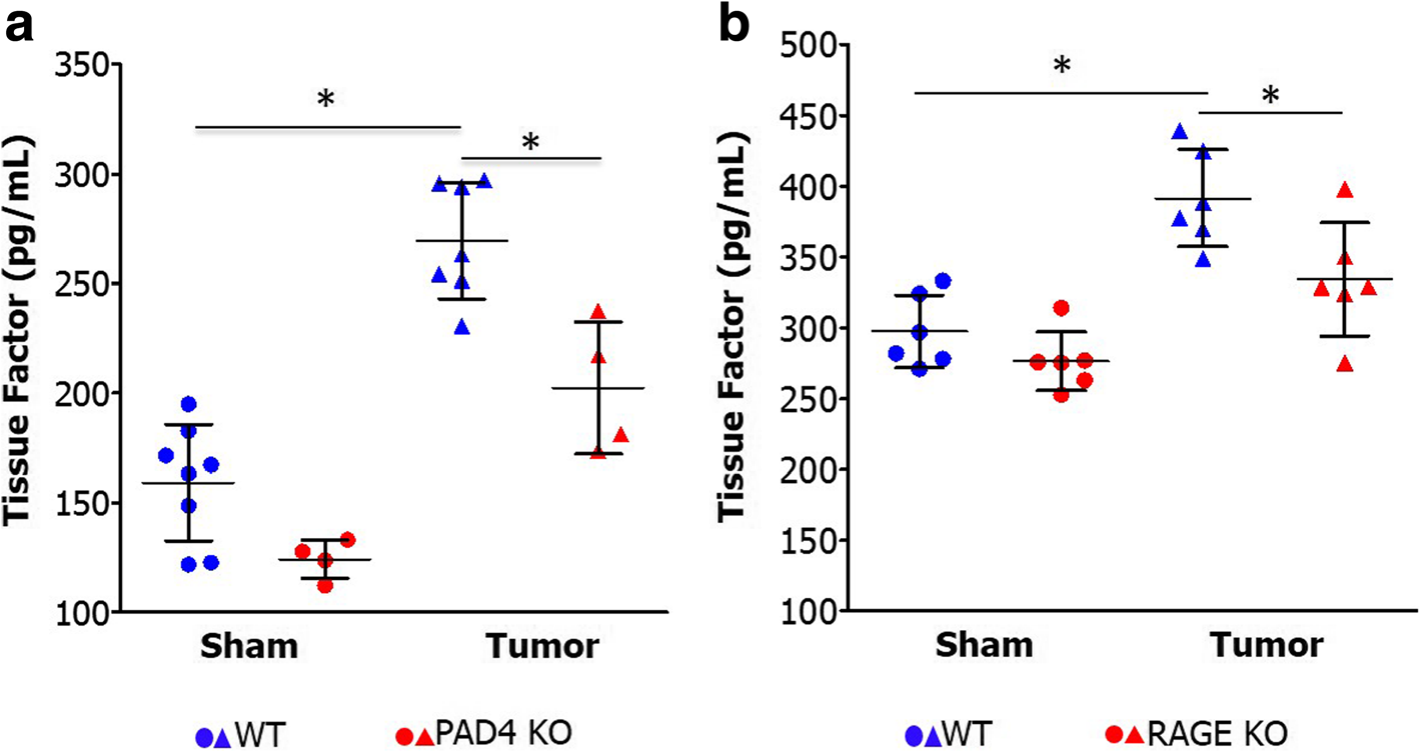
NETs promote hypercoagulability in PDA by releasing circulating tissue factor. Tissue factor ELISA was performed on serum from orthotopic mice, demonstrating that tumor burdened mice had elevated levels of circulating tissue factor compared to sham (**a**, 255 ± 49 vs. 159 ± 26 pg/mL, *p* < 0.05). Genetic deletion of PAD4, thereby inhibiting NET formation, resulted in a substantial decrease in circulating tissue factor levels in tumor bearing mice (269 ± 26 vs. 202 ± 30 pg/mL, *p* < 0.05). Blue = WT, Red = PAD4 KO, Circle = Sham, Triangle = Tumor. RAGE knockout tumor bearing mice, who we have previously shown have decreased NET formation, also had lower levels of tissue factor compared to WT controls (**b**, 331 ± 39 vs. 390 ± 34 pg/mL, *p* < 0.05). *p < 0.05. Blue = WT, Red = RAGE KO, Circle = Sham, Triangle = Tumor

Tissue factor, a transmembrane receptor in subendothelial cells, is a key initiator of the extrinsic coagulation cascade and is a contributor to hypercoagulability in pancreatic cancer [24]. Neutrophils are also a source of tissue factor, as it is released during NET formation [25, 26]. Since NETs are known to release tissue factor, we evaluated levels of circulating tissue factor in our murine models of pancreatic cancer. Tumor bearing mice had elevated levels of serum tissue factor compared with sham controls (Fig. 3a & b). Inhibiting NET formation by genetic depletion of PAD4 resulted in a decrease in serum tissue factor (Fig. 3a). Furthermore, RAGE KO mice, which have diminished NET formation, also had lower levels of serum tissue factor (Fig. 3b).

### Chloroquine decreases NET mediated platelet aggregation and release of tissue factor

**Fig. 4:**
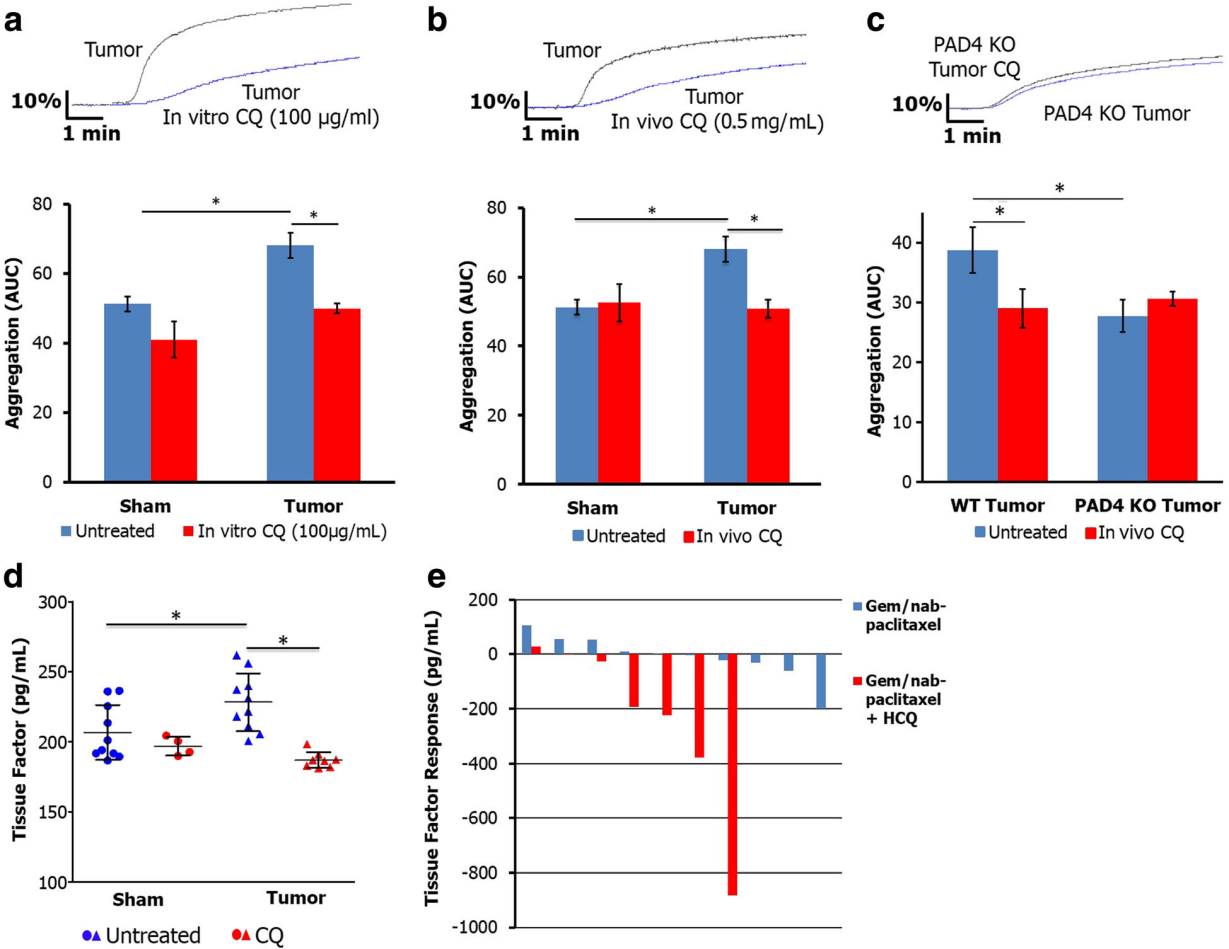
CQ inhibition of NETs reverses platelet aggregation and decreases tissue factor. In vitro treatment of whole blood with CQ led to a significant reduction in platelet aggregation in blood harvested from tumor bearing mice (**a**, AUC 50 ± 2.4 vs. 68.1 ± 8.8, *n* = 4, *p* < 0.05). Treatment of mice with CQ led to a decrease in aggregation in tumor bearing animals with no change in sham (**b**, AUC 52.6 ± 5.3 vs. 68.1 ± 8.8, *n* = 4, *p* < 0.05). Importantly, CQ had minimal effects in PAD4KO mice, suggesting that it decreases platelet aggregation through inhibition of NETs (**c**). CQ treatment led to a decrease in circulating tissue factor in tumor bearing mice (**d**, 186.9 ± 5.6 vs. 228.2 ± 21 pg/mL, *p* < 0.05). Hydroxychloroquine treatment resulted in significant reduction in tissue factor levels in patients with elevated preoperative serum tissue factor compared to control, with a mean response to treatment of − 240 ± 120 versus − 8.74 ± 26 pg/mL (*p* < 0.05, *n* = 10 gem/nab-paclitaxel, *n* = 7 HCQ). Waterfall plot demonstrating individual treatment response to gemcitabine/nab-paclitaxel with and without hydroxychloroquine in patients with elevated preoperative levels (**e**)

Because chloroquine (CQ) inhibits formation of neutrophil extracellular traps [13], we sought to determine if chloroquine treatment would reverse the NET mediated platelet activation and aggregation, and release of tissue factor in tumor bearing animals. Both in vitro treatment of whole blood (Fig. 4a) and in vivo treatment of mice (Fig. 4b) with chloroquine resulted in decreased platelet aggregation and activation (Additional file 2: Figure S2C). To elucidate the potential mechanism of decreased platelet aggregation after CQ treatment, we treated PAD4KO mice with CQ and found that it had minimal effect in these mice, suggesting that CQ mediates decreased platelet aggregation through inhibition of NETs (Fig. 4c). Chloroquine treatment led to a significant reduction in serum tissue factor levels in tumor bearing mice with no significant change in sham mice (Fig. 4d).

We next examined the impact of hydroxychloroquine (HCQ) on circulating tissue factor in patients with pancreatic cancer using serum from our recently completed randomized clinical trial of preoperative gemcitabine/nab-paclitaxel with or without HCQ. There was no difference in pretreatment patient demographics between the two randomized groups (Additional file 4: Table S1). HCQ led to a statistically greater reduction in tissue factor in those patients who had elevated tissue factor prior to treatment, defined by preoperative level greater than the median (40 ng/mL), (− 240 ± 120 vs. -8.74 ± 26.1 pg/mL, *p* < 0.05, Fig. 4e). There was no difference in change in tissue factor with HCQ treatment in those patients with normal pre-treatment levels (mean change with treatment − 55 ± 63 vs. + 3.1 ± 14 pg/mL, *p* = 0.38, *n* = 19 gem/nab-paclitaxel alone, *n* = 18 gem/nab-paclitaxel + HCQ).

### Chloroquine inhibition of NETs reverses hypercoagulability

**Fig. 5:**
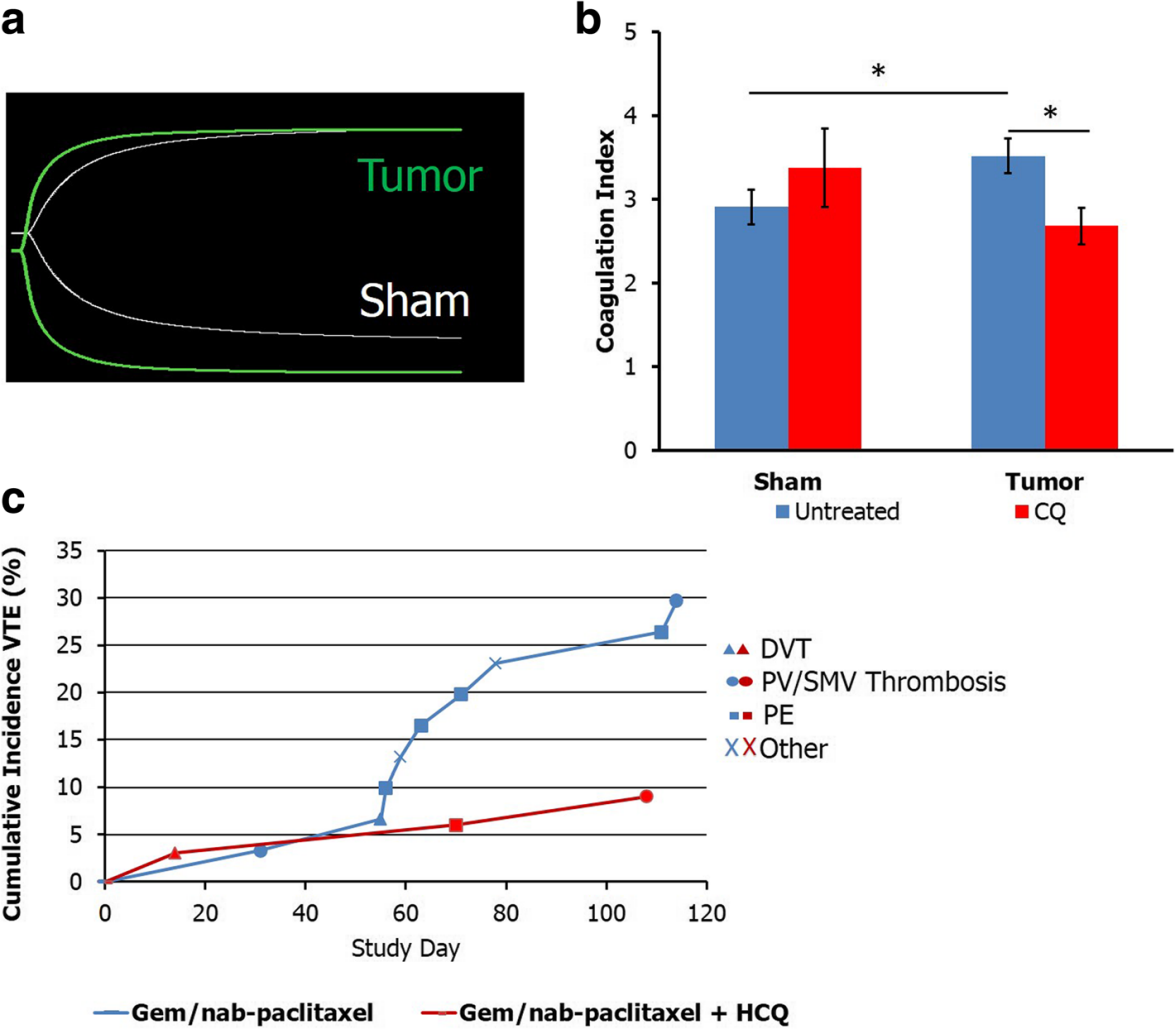
Chloroquine reverses hypercoagulability in pancreatic cancer. Representative TEG curves demonstrating orthotopically injected mice are hypercoagulable compared with sham controls (**a**). Treatment with CQ reverses the hypercoagulability on TEG as measured by coagulation index (**b**). The 90 day VTE rate for patients treated with 2 cycles of preoperative gemcitabine/abraxane + HCQ was 9.1% (*n* = 3 of 33) compared to 30% (*n* = 9 of 30) in patients treated with gemcitabine/abraxane alone (**c**, *p* = 0.053)

To study the effects of chloroquine inhibition of NETs and subsequent decrease in platelet aggregation and circulating tissue factor on the hypercoagulable state seen in pancreatic cancer, we performed thromboelastograms (TEG) in mice with pancreatic adenocarcinoma to assess hypercoagulability as measured by the coagulation index, which takes into account all of the TEG parameters (Additional file 5: Table S2). Tumor mice had an elevated coagulation index compared with sham controls, suggestive of hypercoagulability (Fig. 5a). Treatment with CQ resulted in a decrease in the coagulation index in cancer burdened animals (Fig. 5b).

We next assessed the rate of venous thromboembolism (VTE) in patients treated with pre-operative hydroxychloroquine as part of two separate clinical trial protocols. In patients treated as part of a phase I/II dose escalation trial of preoperative hydroxychloroquine with gemcitabine, the 90 day VTE rate was 3% (n = 1 of 33) [18]. Of note, the lone patient who developed VTE was treated as part of the dose escalation at 800 mg per day rather than at the maximum dose of 1200 mg. In a more recent randomized trial of preoperative gemcitabine and nab-paclitaxel with or without hydroxychloroquine, the VTE rate of patients treated with hydroxychloroquine was 9.1% compared to 30% in patients treated with gemcitabine/nab-paclitaxel alone (*p* = 0.053, Fig. 5c). Mean plasma DNA decreased with treatment in the HCQ group, consistent with potential NET inhibition (601 ± 129 vs. 539 ± 114 ng/mL, *p* < 0.05), but not in the gemcitabine/nab-paclitaxel alone group (588 ± 144 vs. 543 ± 166 ng/mL, *p* = 0.09). Among all patients, those with VTE had a mean increase of 6 ng/mL with treatment compared with decrease of 70 ng/mL in those that did not have VTE (*p* < 0.05). There was a trend towards change in plasma DNA with treatment being associated with development of VTE in patients treated with gemcitabine/nab-paclitaxel alone. Gemcitabine/nab-paclitaxel treated patients who had a VTE had a mean increase of 20 ng/mL following treatment compared with a mean decrease of 76 ng/mL in patients who did not develop VTE (*p* = 0.08). There was no correlation between plasma DNA and VTE in HCQ treated patients.

## Discussion

It has long been recognized that patients with pancreatic cancer are prone to venous thrombosis and it continues to be a major source of morbidity and mortality [27]. After initially being described in sepsis, neutrophil extracellular traps (NETs) were discovered in malignancy and promote tumor growth [28], development of metastases [29, 30] and serve as a potential contributor to cancer associated thrombosis [10]. The current work explores upregulation of platelet function and release of tissue factor as two mechanisms through which NETs contribute to hypercoagulability and thrombosis in pancreatic cancer. Furthermore, because autophagy is critical for NETs in pancreatic cancer, we investigated the use of the autophagy/NET inhibitor chloroquine to reverse NET mediated hypercoagulability in murine models and human patients.

Neutrophil-platelet interactions are increasingly recognized as an important collaboration in promoting malignancy and thrombosis [31]. Activated platelets are capable of inducing NETs [32] and NETs in turn promote platelet aggregation as observed in sepsis and deep vein thrombosis [33, 34]. Cancer induced platelet activation contributes to tumor growth, development of metastases and thrombosis [35, 36]. The current study identifies NETs as a key contributor to platelet aggregation in pancreatic cancer. During NET formation, PAD4 mediated histone citrullination leads to unwinding and release of DNA from neutrophils [37]. Since DNA is known to increase platelet aggregation in sepsis and deep vein thrombosis, we suspected that DNA released during NETosis would also mediate platelet aggregation in pancreatic cancer [33, 34, 38]. Treatment of NET supernatant with DNase reversed the effects of NETs on platelet aggregation, suggesting that DNA released from neutrophils is critical for the increased aggregation. Similarly, Razak et al. also showed that pancreatic cancer NETs promoted platelet adhesion and that these effects could be reversed with DNase [11]. We confirmed these observations and expanded on this mechanism to include the receptor for advanced glycation end products (RAGE), a known receptor for extracellular DNA, as a critical component of NET mediated platelet aggregation in pancreatic cancer. The addition of NET supernatant to RAGE knockout blood did not result in increased platelet aggregation. Additionally, RAGE knockout mice had no differences in platelet aggregation at baseline, but had decreased platelet aggregation in tumor burdened mice compared with wild type. While these findings point to extracellular DNA and RAGE promoting NET mediated platelet aggregation, there are many components released from NETs that may also have an impact on hypercoagulability and were not evaluated in the current analysis.

Tissue factor, a transmembrane receptor typically found in subendothelial cells that binds to factor VII to initiate the extrinsic pathway when the endothelium is damaged is also released from neutrophils during NET formation [25, 26]. Tissue factor thought to be derived from tumor associated microparticles has been linked to pancreatic cancer thrombosis [39–42] and levels of tissue factor predict venous thromboembolism in cancer patients [43]. We identified NETs as a potential source of circulating tissue factor in pancreatic cancer, as genetic deletion of PAD4, an enzyme critical for NET formation, resulted in significant reduction in circulating tissue factor in tumor bearing mice. Importantly, PAD4 also citrullinates and inhibits antithrombin [44, 45], suggesting another possible mechanism of hypercoagulability in pancreatic cancer. This does potentially confound our results in PAD4 knockout mice and must be taken into account when considering our findings.

Because autophagy is critical to the process of NET formation, we studied the novel use of the autophagy inhibitor chloroquine to target NET mediated hypercoagulability. Chloroquine has been used for many years to treat patients with malaria, lupus, and rheumatoid arthritis, but more recently, hydroxychloroquine has been evaluated as a treatment for pancreatic cancer, with encouraging preliminary results [18]. Chloroquine has previously been studied for prevention of perioperative VTE in orthopedic surgery patients, however these studies had mixed results and the precise mechanism was not completely understood [46, 47]. Subsequent studies have established that HCQ has direct effects on platelet activation and aggregation [48, 49]. However, our group and others have demonstrated that chloroquine prevents NET formation [13, 14]; therefore some of the antiplatelet effects of HCQ may be secondary to reduction in NET mediated DNA release which increases platelet aggregation. In the current study, inhibition of NETs with chloroquine resulted in decreased platelet aggregation and lower levels of circulating tissue factor. In patients who had elevated levels of pre-treatment tissue factor, HCQ treatment led to a significant reduction, suggesting that the greatest effect of HCQ is seen in patients who may have upregulation of NETs at baseline. Based on this data, inhibition of NET formation may also explain the previously recognized reduction in VTE rate. Importantly, treatment with CQ in PAD4 KO mice, incapable of forming NETs, had minimal effect, suggesting that CQ decreases platelet aggregation through inhibition of NETs. However, because CQ also has direct antiplatelet effects, it is difficult to completely attribute all its effects to inhibition of NETosis.

Traditional coagulation tests such as prothrombin time (PT), partial thromboplastin time (PTT), and international normalized ratio (INR) are frequently normal in hypercoagulability and provide limited information regarding the mechanisms driving a prothrombotic state. To study the role of chloroquine inhibition of NETs and hypercoagulability using a more informative and clinically translatable approach, we utilized thromboelastograms to evaluate whether treatment with chloroquine decreases hypercoagulability in orthotopic murine pancreatic cancer. TEG has been most thoroughly studied in patients during massive bleeding from trauma as a rapidly available test to direct transfusion of blood products, however, it is becoming more frequently utilized to identify hypercoagulability [20]. Hypercoagulable changes are detectable on rotational thromboelastometry, similar to TEG, in patients with abdominal malignancy [50]. We demonstrate that tumor burdened mice are hypercoagulable on TEG and treatment with chloroquine reverses this hypercoagulopathy. Importantly, control sham mice appear to have a subtle increase in coagulation index with CQ treatment. It is possible that CQ may only serve a beneficial role in reducing hypercoagulability in the cancer burdened state, where NETs are upregulated. This could explain why prior randomized trials of CQ to decrease VTE in non-malignant orthopedic patients were inconclusive [46, 47].

Given its well-established use, favorable safety profile and anti-tumor effects, CQ is a suitable treatment to decrease VTE rate in patients with pancreatic cancer. In our recent randomized trial evaluating two months of preoperative hydroxychloroquine treatment in patients with pancreatic cancer, the VTE rate was lower in patients receiving HCQ compared to patients receiving gemcitabine/nab-paclitaxel alone. Although designed and powered to study the effects of HCQ on pathologic treatment response and decrease in Ca 19–9, the reduction in VTE rate neared statistical significance. Additionally, the 90 day postoperative reduction in VTE occurred despite HCQ stopping at time of surgery. We identified a trend towards an increase in plasma DNA with treatment and development of VTE, which has been previously recognized as a marker for risk of VTE [51]. DNA is released from neutrophils into the circulation during NET formation, therefore this data suggests that NETs may play a role in VTE in patients with pancreatic cancer. However, given that DNA is a nonspecific marker for NETs and that circulating DNA in cancer patients is likely derived from multiple sources [52] we are unable to conclude that DNA released from NETs is driving VTE in these patients. Nonetheless, these findings support a clinical trial designed specifically to study reduction in VTE by treatment of cancer patients with perioperative HCQ.

## Conclusion

We demonstrate in murine models of pancreatic cancer that NETs promote hypercoagulability by increasing platelet aggregation through DNA release and RAGE as well as by release of tissue factor. Treatment with the autophagy inhibitor chloroquine results in a reversal of hypercoagulability in pancreatic cancer by diminishing NET mediated platelet aggregation and release of circulating tissue factor and improving coagulation index on TEG. We have for the first time also provided evidence that these pathways play a role in human pancreatic cancer. All together our findings support additional clinical trials with hydroxychloroquine to examine the ability of NET inhibition to lower the venous thromboembolism rate in patients with pancreatic and other cancer types.

## Additional files

Additional file 1: **Figure S1.** Formation of ex vivo NETs. Microscopy of isolated neutrophils stimulated with platelet activating factor (PAF) and stained with Hoechst to visualize extracellular DNA, demonstrating ex vivo neutrophil extracellular trap (NET) formation. (DOCX 221 kb)

Additional file 2: **Figure S2.** Neutrophil Extracellular Traps (NETs) promote platelet activation in murine pancreatic adenocarcinoma. Platelet activation was assessed by measuring % CD62P positive cells by flow cytometry. Tumor burdened mice had heightened platelet activation compared to sham controls (A). PAD4 KO mice, unable to form NETs had diminished platelet activation. Addition of NET supernatant to murine whole blood increased platelet activation in a dose dependent fashion (B). Chloroquine treatment reversed the tumor associated increase in platelet activation (C). (DOCX 109 kb)

Additional file 3: **Figure S3.** Neutrophil and fibrinogen conjugates in the pancreatic tumor microenvironment. Pancreatic tumor specimens from resected patients with pancreatic adenocarcinoma were stained for neutrophil elastase (red) and fibrinogen (white). Representative images from three individual patients are shown, demonstrating focal areas of elastase and fibrinogen in the tumor, suggesting interactions between neutrophils and thrombosis in the tumor microenvironment. (DOCX 489 kb)

Additional file 4: **Table S1.** Select results of randomized trial of potentially resectable pancreatic cancer patients treated with preoperative gemcitabine/nab-paclitaxel with and without hydroxychloroquine (HCQ). There were no significant differences in pretreatment patient demographics or characteristics. Correlative markers of NET formation including circulating levels of DNA and tissue factor were also assessed as discussed in the manuscript. Pre-tx = Pre-treatment, CCI=Charlson Comorbidity Index, EUS = Endoscopic ultrasound. (DOCX 15 kb)

Additional file 5: **Table S2.** CQ reverses hypercoagulability in tumor burdened mice. Thromboelastogram (TEG) values for orthotopic tumor and sham mice with and without chloroquine (CQ) treatment, demonstrating that tumor mice have hypercoagulable elevations in K, angle, maximum amplitude (MA) and coagulation index (CI) compared with sham controls and that CQ reverses hypercoagulability as assessed by the CI. *p < 0.05 vs. Sham, **p < 0.05 vs. Tumor. (DOCX 14 kb)

## Abbreviations

AUC: Area under the curve
CQ: Chloroquine
HBSS: Hank’s balanced salt solution
HCQ: Hydroxychloroquine
HMGB1: High mobility group box 1
IRB: Institutional Review Board
NET: Neutrophil extracellular trap
PAD 4: Protein arginine deiminase 4
PDA: Pancreatic ductal adenocarcinoma
PMA: Phorbol 12-myristate 13-acetate
RAGE: Receptor for advanced glycation end products
TEG: Thromboelastogram
VTE: Venous thromboembolism
