# Supplementary material for: Chloroquine reduces hypercoagulability in pancreatic cancer through inhibition of neutrophil extracellular traps
Source: BMC Cancer. 2018 Jun 22;18:678. doi: 10.1186/s12885-018-4584-2 (PMC6013899; doi:10.1186/s12885-018-4584-2)
Supplement: Supplementary file 1 — Figure S1. Formation of ex vivo NETs. Microscopy of isolated neutrophils stimulated with platelet activating factor (PAF) and stained with Hoechst to visualize extracellular DNA, demonstrating ex vivo neutrophil extracellular trap (NET) formation. (DOCX 221 kb) [file 12885_2018_4584_MOESM1_ESM.docx]

**
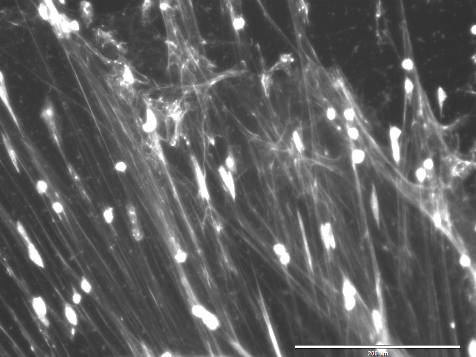
**

**Figure S1: Formation of *ex vivo* NETs.** Microscopy of isolated neutrophils stimulated with platelet activating factor (PAF) and stained with Hoechst to visualize extracellular DNA, demonstrating *ex vivo* neutrophil extracellular trap (NET) formation.
