# Supplementary material for: Chloroquine reduces hypercoagulability in pancreatic cancer through inhibition of neutrophil extracellular traps
Source: BMC Cancer. 2018 Jun 22;18:678. doi: 10.1186/s12885-018-4584-2 (PMC6013899; doi:10.1186/s12885-018-4584-2)
Supplement: Supplementary file 2 — Figure S2. Neutrophil Extracellular Traps (NETs) promote platelet activation in murine pancreatic adenocarcinoma. Platelet activation was assessed by measuring % CD62P positive cells by flow cytometry. Tumor burdened mice had heightened platelet activation compared to sham controls (A). PAD4 KO mice, unable to form NETs had diminished platelet activation. Addition of NET supernatant to murine whole blood increased platelet activation in a dose dependent fashion (B). Chloroquine treatment reversed the tumor associated increase in platelet activation (C). (DOCX 109 kb) [file 12885_2018_4584_MOESM2_ESM.docx]

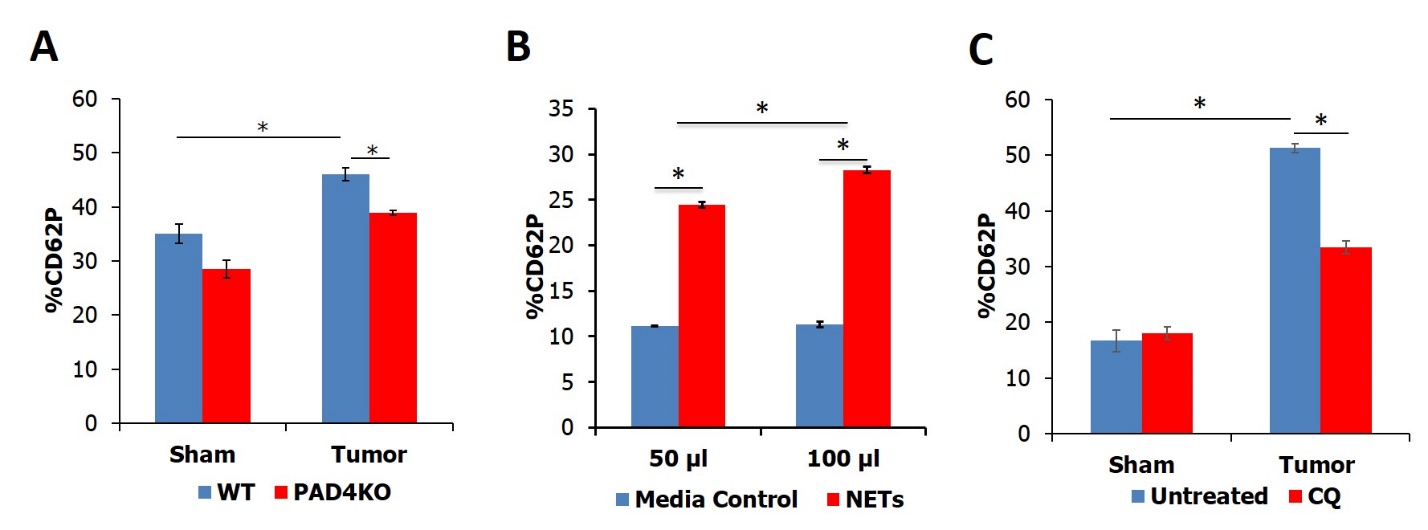
**Figure S2: Neutrophil Extracellular Traps (NETs) promote platelet activation in murine pancreatic adenocarcinoma**. Platelet activation was assessed by measuring % CD62P positive cells by flow cytometry. Tumor burdened mice had heightened platelet activation compared to sham controls (A). PAD4 KO mice, unable to form NETs had diminished platelet activation. Addition of NET supernatant to murine whole blood increased platelet activation in a dose dependent fashion (B). Chloroquine treatment reversed the tumor associated increase in platelet activation (C).
