# Supplementary material for: Chloroquine reduces hypercoagulability in pancreatic cancer through inhibition of neutrophil extracellular traps
Source: BMC Cancer. 2018 Jun 22;18:678. doi: 10.1186/s12885-018-4584-2 (PMC6013899; doi:10.1186/s12885-018-4584-2)
Supplement: Supplementary file 3 — Figure S3. Neutrophil and fibrinogen conjugates in the pancreatic tumor microenvironment. Pancreatic tumor specimens from resected patients with pancreatic adenocarcinoma were stained for neutrophil elastase (red) and fibrinogen (white). Representative images from three individual patients are shown, demonstrating focal areas of elastase and fibrinogen in the tumor, suggesting interactions between neutrophils and thrombosis in the tumor microenvironment. (DOCX 489 kb) [file 12885_2018_4584_MOESM3_ESM.docx]

**
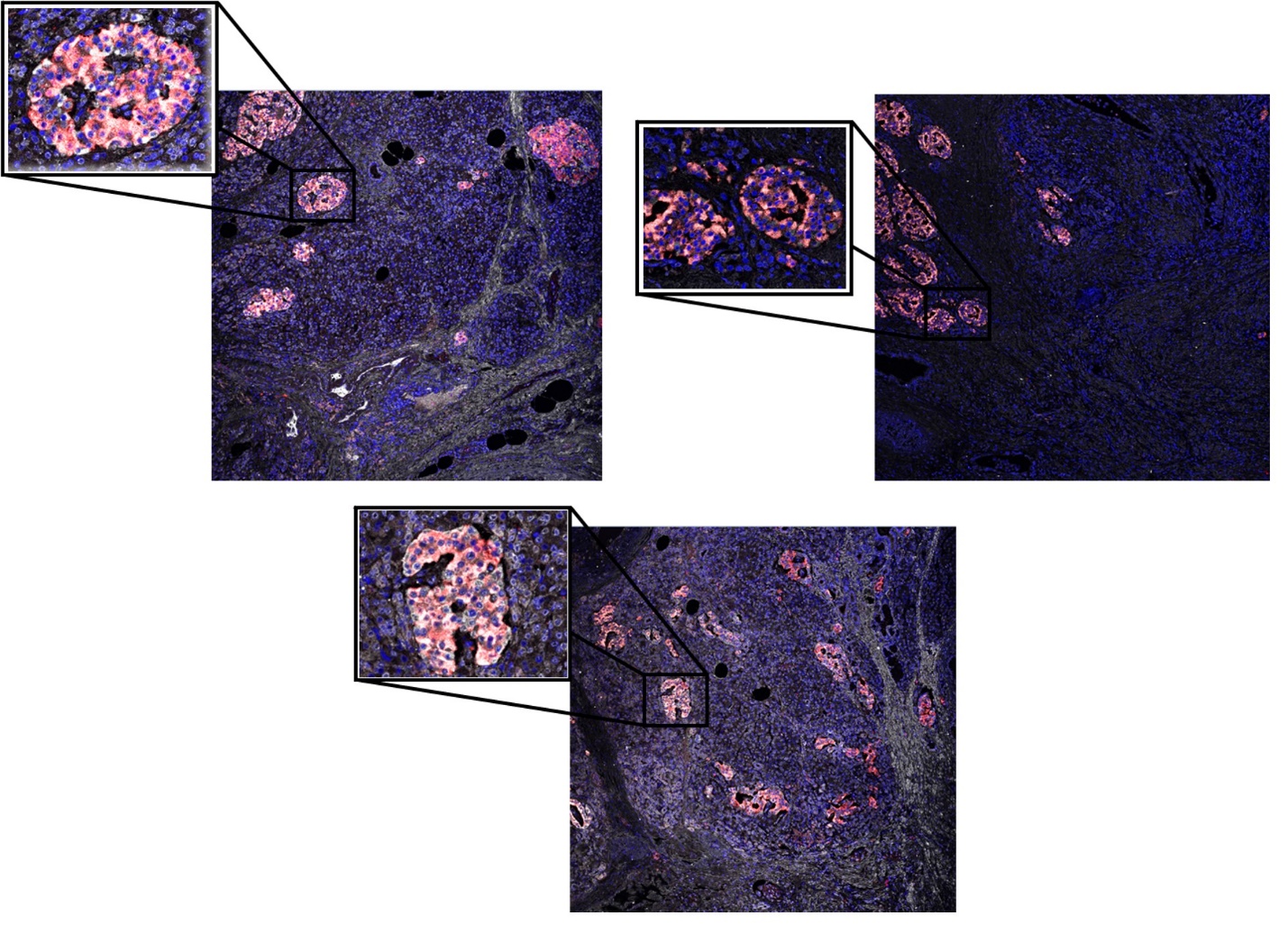
**

**Figure S3: Neutrophil and fibrinogen conjugates in the pancreatic tumor microenvironment.** Pancreatic tumor specimens from resected patients with pancreatic adenocarcinoma were stained for neutrophil elastase (red) and fibrinogen (white). Representative images from three individual patients are shown, demonstrating focal areas of elastase and fibrinogen in the tumor, suggesting interactions between neutrophils and thrombosis in the tumor microenvironment.
