# Supplementary material for: Chloroquine reduces hypercoagulability in pancreatic cancer through inhibition of neutrophil extracellular traps
Source: BMC Cancer. 2018 Jun 22;18:678. doi: 10.1186/s12885-018-4584-2 (PMC6013899; doi:10.1186/s12885-018-4584-2)
Supplement: Supplementary file 4 — Table S1. Select results of randomized trial of potentially resectable pancreatic cancer patients treated with preoperative gemcitabine/nab-paclitaxel with and without hydroxychloroquine (HCQ). There were no significant differences in pretreatment patient demographics or characteristics. Correlative markers of NET formation including circulating levels of DNA and tissue factor were also assessed as discussed in the manuscript. Pre-tx = Pre-treatment, CCI=Charlson Comorbidity Index, EUS = Endoscopic ultrasound. (DOCX 15 kb) [file 12885_2018_4584_MOESM4_ESM.docx]

|  |  | **Gem/nab-paclitaxel (n=30)** | **Gem/nab-paclitaxel + HCQ (n=33)** | **p** |
| --- | --- | --- | --- | --- |
| **Patient Characteristics** | |  |  |  |
|  | **Age** (median) | 64 | 67 | 0.4 |
|  | **Male,** n(%) | 16 (53%) | 18 (54%) | 1.0 |
|  | **CCI** (mean) | 4.6 | 4.8 | 0.56 |
|  | **Pre-tx aspirin, n(%)** | 8 (27%) | 9 (27%) | 1.0 |
|  | **Pre-tx anticoagulation, n(%)** | 1 (3%) | 3 (9%) | 0.61 |
|  | **EUS** **Tumor Size** (cm) | 2.6 | 2.8 | 0.31 |
|  | **EUS Node +**, n(%) | 19 (63%) | 19 (58%) | 0.80 |
|  | **Vein Resection**, n(%) | 16 (53%) | 13 (39%) | 0.32 |
|  | **Estimated Blood Loss** (median, mL) | 163 | 150 | 0.41 |

**Table S1: Select results of randomized trial of potentially resectable pancreatic cancer patients treated with preoperative gemcitabine/nab-paclitaxel with and without hydroxychloroquine (HCQ).** There were no significant differences in pretreatment patient demographics or characteristics. Correlative markers of NET formation including circulating levels of DNA and tissue factor were also assessed as discussed in the manuscript. Pre-tx= Pre-treatment, CCI=Charlson Comorbidity Index, EUS= Endoscopic ultrasound.
