# Supplementary material for: Chloroquine reduces hypercoagulability in pancreatic cancer through inhibition of neutrophil extracellular traps
Source: BMC Cancer. 2018 Jun 22;18:678. doi: 10.1186/s12885-018-4584-2 (PMC6013899; doi:10.1186/s12885-018-4584-2)
Supplement: Supplementary file 5 — Table S2. CQ reverses hypercoagulability in tumor burdened mice. Thromboelastogram (TEG) values for orthotopic tumor and sham mice with and without chloroquine (CQ) treatment, demonstrating that tumor mice have hypercoagulable elevations in K, angle, maximum amplitude (MA) and coagulation index (CI) compared with sham controls and that CQ reverses hypercoagulability as assessed by the CI. *p < 0.05 vs. Sham, **p < 0.05 vs. Tumor. (DOCX 14 kb) [file 12885_2018_4584_MOESM5_ESM.docx]

| **Group** | **R (min)** | **K (min)** | **Angle (°)** | **MA (mm)** | **CI** |
| --- | --- | --- | --- | --- | --- |
| **Sham** | 3.3 ±1.1 | 2.6 ± 1.2 | 58.6 ± 9.5 | 60.7 ± 6.1 | 2.9 ± 0.7 |
| **Tumor** | 3.0 ± 1.6 | 1.5 ± 0.6* | 69.8 ± 7.6* | 65.7 ± 5.3* | 3.5 ± 0.7* |
| **Sham + CQ** | 3.5 ± 1.0 | 1.6 ± 0.5 | 66.4 ± 6.0 | 65.5 ± 6.0 | 3.4 ± 1.0 |
| **Tumor + CQ** | 4.2 ± 2.4 | 1.8 ± 0.5 | 66.1 ± 4.7 | 62.2 ± 3.1 | 2.7 ± .5** |

**Table S2: CQ reverses hypercoagulability in tumor burdened mice.** Thromboelastogram (TEG) values for orthotopic tumor and sham mice with and without chloroquine (CQ) treatment, demonstrating that tumor mice have hypercoagulable elevations in K, angle, maximum amplitude (MA) and coagulation index

(CI) compared with sham controls and that CQ reverses hypercoagulability as assessed by the CI. *p<0.05 vs. Sham, ** p<0.05 vs. Tumor.
